# Accurate Modelling of AFM Force-Indentation Curves with Blunted Indenters at Small Indentation Depths

**DOI:** 10.3390/mi15101209

**Published:** 2024-09-29

**Authors:** Stylianos Vasileios Kontomaris, Anna Malamou, Andreas Stylianou

**Affiliations:** 1 Cancer Mechanobiology and Applied Biophysics Group, School of Sciences, European University Cyprus, 2404 Nicosia, Cyprus; stylianou.c.andreas.1@ucy.ac.cy; 2 School of Electrical and Computer Engineering, National Technical University of Athens, 15773 Athens, Greece; annamalamou@yahoo.gr

**Keywords:** sphero-conical indenter, biological materials, blunt pyramid, Young’s modulus, data processing, mathematical modelling, dimensional analysis

## Abstract

When testing biological samples with atomic force microscopy (AFM) nanoindentation using pyramidal indenters, Sneddon’s equation is commonly used for data processing, approximating the indenter as a perfect cone. While more accurate models treat the AFM tip as a blunted cone or pyramid, these are complex and lack a direct relationship between applied force and indentation depth, complicating data analysis. This paper proposes a new equation derived from simple mathematical processes and physics-based criteria. It is accurate for small indentation depths and serves as a viable alternative to complex classical approaches. The proposed equation has been validated for ℎ < 3*R* (where h is the indentation depth and R is the tip radius) and confirmed through simulations with blunted conical and pyramidal indenters, as well as experiments on prostate cancer cells. It is a reliable method for experiments where the tip radius cannot be ignored, such as in shallow indentations on thin samples to avoid substrate effects.

## 1. Introduction

Atomic force microscopy (AFM) is an extremely effective method for nanoscale imaging and mechanical characterization of soft materials [1,2]. By applying the AFM nanoindentation technique, Young’s modulus maps of biological materials can be produced, aiding in the diagnosis of various diseases [2,3]. AFM is not the only ultrasensitive tool for determining the nanomechanical properties of soft materials. Other techniques, such as ultrasound vibrometry and optical tweezers, also exist [4]. Specifically, ultrasound vibrometry enables the noninvasive measurement of shear wave propagation velocity in soft tissue, allowing for the inverse estimation of tissue shear moduli across multiple frequencies. Mathematical methods and model behavior analysis can be used to select the appropriate model for tissue characterization [4]. Optical tweezers are a powerful technique that uses a highly focused laser beam to trap objects [5,6]. They are used to apply forces or torques to individual molecules. Coated plastic beads and soft matter materials can experience underlying attractive or repulsive forces in the piconewton range [5,6]. Compared to ultrasound vibrometry, AFM provides nanometer-scale spatial resolution, making it ideal for analyzing the nanomechanical properties of materials at very small scales (down to individual molecules or cells). Ultrasound vibrometry, on the other hand, is more suited to larger-scale tissue measurements and lacks such fine resolution. In other words, ultrasound vibrometry is mostly effective at assessing bulk mechanical properties and is less sensitive to surface-specific details. In comparison to optical tweezers, AFM can measure a much wider range of forces, from piconewtons (pN) to micronewtons (µN), making it suitable for various materials, from very soft to very hard. Optical tweezers are typically limited to measuring forces in the piconewton range, making them more suitable for softer materials or delicate biological systems, such as individual molecules or cells.

Notably, groundbreaking studies have shown that the AFM nanoindentation technique can differentiate between normal and cancerous cells [7]; classify human tissues as normal, benign, or malignant [8,9]; assist in the early diagnosis of osteoarthritis [10]; and allow for the mechanical characterization of proteins [11,12] and viruses [13], among other applications. The main benefit of this approach is its capability to diagnose diseases autonomously, relying on mathematical criteria and automated computational methods. However, several challenges must be overcome before AFM technology can be fully integrated into clinical practice [14]. The main objective of AFM research on biological materials is to develop a reliable system for nanoscale characterization of biological materials, such as cells and tissues, for medical purposes. Achieving this requires the development of intelligent systems that minimize complexity and experimental effort. When testing soft samples at the nanoscale using pyramidal indenters, the most common approach is to approximate the indenter as a conical shape [15,16,17,18,19] and fit the data to the following equation:

(1) $$F=\frac{2}{\pi }\frac{E}{1-v^{2}}\tan\left(\theta \right)\;h^{2}\;$$

where F is the applied force, h is the indentation depth, and E and ν are the Young’s modulus and Poisson’s ratio of the tested material, respectively. In addition, θ is the half-angle of the conical indenter. However, the most accurate approximation of the shape of real pyramidal indenters is either a sphero-conical shape or a blunt pyramid [20,21]. Despite the existence of accurate mathematical expressions for these cases [20,21], data processing is not as straightforward as it is for the case of Equation (1). The reason is that they do not provide a direct relationship between the applied force and the indentation depth. Therefore, these accurate equations are often omitted when testing soft biological samples. Equation (1) is valid when the indentation depth is significantly larger than the indenter’s apex radius R, i.e., h >> R. A simplified approach was recently presented for large sphero-conical indentations; however, it was designed for h > 3R, as it approximates the transition depth between the spherical and conical parts of the indenter with h_T_ ≈ R [22]. Therefore, a critical question arises: Is it possible to simplify data processing for indenters with a non-negligible tip apex radius at any indentation depth? More specifically, the aim is to fit the data to a straightforward equation of the following form:

(2) $$F=ch^{m}\;$$

Subsequently, the Young’s modulus should be calculated using the fitting parameter c. Equation (1) is a subcase of Equation (2) in which m=2 and $c=\frac{2}{\pi }\frac{E}{1-v^{2}}\tan\left(\theta \right)$. 

This paper is organized as follows: Section 2 (Materials and Methods) presents the equations that best approximate real pyramidal indenters and introduces a novel mathematical analysis to demonstrate that a simple equation, as given in Equation (2), can yield accurate results. Section 3 (Results) processes simulated force-indentation curves using both the classic equations for blunted conical or blunted pyramidal indenters and the new simplified approach, with the latter providing accurate results. Force-indentation data from prostate cancer cells were also processed using both the classic and the newly proposed methods. Section 4 (Discussion) offers additional clarifications on the proposed method and its limitations, including a discussion of its application to highly heterogeneous materials.

## 2. Materials and Methods

### 2.1. Equations for Approximating the Behavior of Real Indenters

The primary goal of this section is to derive a simple equation, in the form of Equation (2), that relates the applied force on the sample to the indentation depth and the sample’s Young’s modulus. The accurate force-indentation relationship for a sphero-conical indenter is provided below [20]:

(3) $$F=\frac{2E}{1-v^{2}}\left\{ah-\frac{a^{2}}{2\tan\left(\theta \right)}\left[\frac{\pi }{2}-\arcsin\left(\frac{b}{a}\right)\right]-\frac{a^{3}}{3R}+{\left(a^{2}-b^{2}\right)}^{1/2}\left(\frac{b}{2\tan(\theta )}+\frac{\alpha^{2}-b^{2}}{3R}\right)\right\}\;$$

In Equation (3), *E* and *ν* are the Young’s modulus and Poisson’s ratio of the tested sample, respectively; *θ* is the cone’s semi-angle; *R* is the radius of the tip apex; *α* is the contact radius (i.e., the radius at contact depth); and *b* is the radius at the transition depth between the spherical and conical parts of the indenter [20]. Equation (3) is valid for a>b [20]. When the spherical tip merges seamlessly (tangentially) with the body of the cone,

(4) $$b=Rcos\left(\theta \right)\;$$

In addition, the contact depth is related to the contact radius by the following equation [20]:

(5) $$h+\frac{a}{R}\left[{\left(a^{2}-b^{2}\right)}^{1/2}-a\right]-\frac{a}{\tan\left(\theta \right)}\left[\frac{\pi }{2}-\arcsin\left(\frac{b}{a}\right)\right]=0\;$$

Equations (3)–(5) are accurate; however, they have two major disadvantages. The first is that they do not directly relate the applied force to the indentation depth, which makes data processing challenging. The second is that they cannot be used to fit the entire range of data. In particular, for small indentation depths (i.e., before the transition to the conical part of the indenter (a<b)), the parameter ${\left(a^{2}-b^{2}\right)}^{1/2}$ is not a real number. The same limitations also apply to blunted pyramidal indenters [21]. Consider a blunted k-sided rigid regular pyramid of semi-included angle θ and spherical cap radius R. In this case, the applied force is given by the equation below [21]:

(6) $$F=\frac{2E}{1-v^{2}}\left\{ah-\frac{k}{\pi }sin\left(\frac{\pi }{k}\right)\frac{a^{2}}{2\tan\left(\theta \right)}\left[\frac{\pi }{2}-\arcsin\left(\frac{b}{a}\right)\right]-\frac{a^{3}}{3R}+{\left(a^{2}-b^{2}\right)}^{1/2}\left(\frac{k}{\pi }sin\left(\frac{\pi }{k}\right)\frac{b}{2\tan(\theta )}+\frac{\alpha^{2}-b^{2}}{3R}\right)\right\}\;$$

and

(7) $$h+\frac{a}{R}\left[{\left(a^{2}-b^{2}\right)}^{1/2}-a\right]-\frac{a}{\tan\left(\theta \right)}\frac{k}{\pi }sin\left(\frac{\pi }{k}\right)\left[\frac{\pi }{2}-\arcsin\left(\frac{b}{a}\right)\right]=0\;$$

It should be also noted that for both cases (sphero-conical or blunted pyramid), for a<b, the force-indentation relationship is the simple Hertzian equation [20,21]:

(8) $$F=\frac{4}{3}\frac{E}{1-v^{2}}R^{1/2}h^{3/2}\;$$

On the other hand, Sneddon derived a general equation for any axisymmetric indenter that correlates the applied force with the indentation depth [23,24]:

(9) $$F=\frac{2E}{{\left(\sqrt{\pi }Β\right)}^{\frac{1}{n}}(1-v^{2})}\left(\frac{n}{n+1}\right){\left[\frac{\Gamma \left(\frac{n}{2}+\frac{1}{2}\right)}{\Gamma \left(\frac{n}{2}+1\right)}\right]}^{\frac{1}{n}}h^{1+\frac{1}{n}}\;$$

where Γ represents the gamma function and B, n are constants that depend on the indenter’s geometry. The parameter n is related to the exponent m of Equation (2) as follows [23]:

(10) $$m=1+\frac{1}{n}\;$$

For parabolic indenters n=2 (m=3/2), while for perfect conical indenters n=1 (m=2). In addition, for parabolic indenters B=1/(2R) and for conical indenters B=1/tan⁡(θ) [24]. Equation (9) can be used instead of Equations (3)–(5) or (6) and (7). However, there is a major restriction. Since the shape of the indenter in contact with the sample changes with indentation depth, it is impossible to calculate Young’s modulus using Equation (9) because *B* is an unknown, depth-dependent parameter.

### 2.2. Deriving a Simple Equation for Real Indenters

As already mentioned, for real indenters, the parameter *B* is strongly depth-dependent. When approximating a real indenter as sphero-conical, the parameter B should be B=1/(2R) for h≪R (parabolic approximation) and B=1/tan⁡(θ) for h≫R (conical approximation). It is interesting to note that the parameter *B* not only changes its value but also its units. For h≪R it is measured in ${meters}^{-1}$, while for h≫R is dimensionless. Therefore, the idea is to approximate the parameter $B^{1/n}$ with a function that depends on R, θ, n as follows:

(11) $$B^{1/n}=(2{R)}^{\frac{1}{n}-1}\cdot {\left[\tan\left(\theta \right)\right]}^{n-2}/\;f(n)\;$$

where f(n) is a dimensionless correction factor that depends only on the parameter n and $f\left(1\right)=f\left(2\right)=1.$ Hence, Equation (9) is approximated to the following one:

(12) $$F=\frac{2E}{{\left(\sqrt{\pi }\right)}^{\frac{1}{n}}(1-v^{2})}(2{R)}^{1-\frac{1}{n}}\cdot {\left[\tan\left(\theta \right)\right]}^{2-n}\;f(n)\left(\frac{n}{n+1}\right){\left[\frac{\Gamma \left(\frac{n}{2}+\frac{1}{2}\right)}{\Gamma \left(\frac{n}{2}+1\right)}\right]}^{\frac{1}{n}}h^{1+\frac{1}{n}}\;$$

The rationale behind Equation (12) is simple. $B^{1/n}$ should be chosen such that n=1 and n=2 in Equation (9), identical to Equations (1) and (8), respectively. In particular, for n=1,

(13) $$F=\frac{E}{(1-v^{2})\sqrt{\pi }}\tan\left(\theta \right)\frac{\Gamma \left(1\right)}{\Gamma \left(\frac{3}{2}\right)}h^{2}=\frac{2}{\pi }\frac{E}{1-v^{2}}\tan\left(\theta \right)h^{2}\;$$

and for n=2,

(14) $$F=\frac{2}{3}\frac{2E}{{\left(\sqrt{\pi }\right)}^{\frac{1}{2}}(1-v^{2})}(2{R)}^{\frac{1}{2}}{\left[\frac{\Gamma \left(\frac{3}{2}\right)}{\Gamma \left(2\right)}\right]}^{\frac{1}{2}}h^{\frac{3}{2}}=\frac{4}{3}\frac{E}{1-v^{2}}R^{1/2}h^{3/2}\;$$

In addition, Equation (11) was chosen to ensure dimensional consistency. More specifically, the units of the parameter $(2{R)}^{1-\frac{1}{n}}\cdot E\cdot h^{1+\frac{1}{n}}$ are

(15) $${\left(meters\right)}^{1-\frac{1}{n}}\times \frac{Newtons}{{\left(meters\right)}^{2}}\times {\left(meters\right)}^{1+\frac{1}{n}}=Newtons\;$$

The only remaining parameter is the correction factor f(n). The simplest choice is the following:

(16) $$f\left(n\right)=n^{2-n}\;$$

Equation (16) satisfies the condition $f\left(1\right)=f\left(2\right)=1$. Using Equations (12) and (16), the parameter *c* in Equation (2) is equal to

(17) $$c=\frac{2E}{{\left(\sqrt{\pi }\right)}^{\frac{1}{n}}\left(1-v^{2}\right)}(2{R)}^{1-\frac{1}{n}}\cdot {\left[n\cdot \tan\left(\theta \right)\right]}^{2-n}\;\left(\frac{n}{n+1}\right){\left[\frac{\Gamma \left(\frac{n}{2}+\frac{1}{2}\right)}{\Gamma \left(\frac{n}{2}+1\right)}\right]}^{\frac{1}{n}}$$

**Figure 1:**
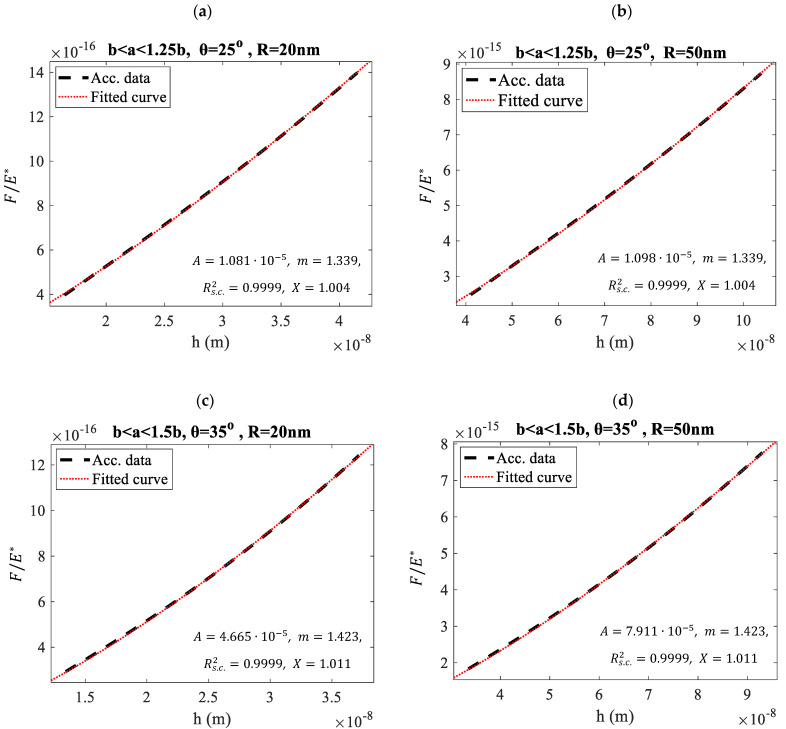
Comparing Equations (3)–(5) with Equations (18) and (19) across various domains. The term ‘Acc. data’ refers to ‘accurate data,’ meaning the plot of the classic Equations (3)–(5), while ‘Fitted curve’ represents Equation (18). The force-indentation data for a theoretical case in the domain b < a < 1.25b use a sphero-conical indenter with $\theta =25^{{}^{\circ}}$ and (**a**) R = 20 nm (18.1 nm<a<22.7 nm), (**b**) R = 50 nm (45.3 nm<a<56.6 nm). In addition, the force-indentation data for a theoretical case in the domain b < a < 1.5b using a sphero-conical indenter with $\theta =35^{{}^{\circ}}$ are also presented. In (**c**), R = 20 nm (16.4 nm<a<24.6 nm), while in (**d**), R = 50 nm (41.0 nm<a<61.4 nm).

Characteristic examples of force-indentation graphs using Equations (3)–(5), and Equations (2), (10), (17) for different domains, using sphero-conical indenters with various geometries, are presented for comparison in Figure 1. In Figure 1a, the domain b<a<1.25b is used, where R=20 nm, $\theta =25^{{}^{\circ}}$ and $b=Rcos\left(\theta \right)≅18\;nm$. The indentation depth for each *a*-value was calculated using Equation (5). The graph $\frac{F}{E^{*}}=f(h)$, where $E^{*}=E/(1-v^{2})$ was plotted using (3)–(5). The data were then fitted to the following equation:

(18) $$y=Ah^{m}$$

where, $y=F/E^{*}$ and A, m are fitting constants. The fitting coefficients were $A=1.081\cdot 10^{-5}$ and m=1.339. The fit was accurate since the R-squared coefficient resulted close to 1 ($R_{s.c.}^{2}=0.9999$). Το check the accuracy of the Young’s modulus calculation we calculated the following ratio:

(19) $$X=\frac{AE^{*}}{c}\;$$

where c is given by Equation (17) and n is calculated using Equation (10). Ideally, the X-ratio should equal to 1. However, Equation (11) is an approximation; therefore, small deviations from the value X = 1 are expected. In particular, the X-value was 1.004, indicating a 0.4% difference. This is a good result that proves the validity of Equations (11) and (17). Three additional examples are also presented. In Figure 1b, the same domain and cone’s semi-angle were used. However, the tip radius was set to 50 nm. In this case as well, X=1.004. In addition, the cases of a sphero-conical indenter with $\theta =35^{{}^{\circ}}$ for indentation in the domain b < a < 1.5b and R = 20 nm (Figure 1c) and R = 50 nm (Figure 1d) are also presented. In these cases, X=1.011. It is also important to note that the cone half-angles of 25° and 35°, along with tip radii of 20 nm and 50 nm, are typical values commonly used in manufactured AFM tips, such as the MLCT-Bio AFM tips produced by Bruker (Billerica, MA, USA). In addition, it is important to note that Equation (17) can also be used instead of Equations (6) and (7), as real pyramidal indenters can be analyzed in terms of an ‘equivalent’ conical or sphero-conical indenter [22].

### 2.3. Open-Access Simulated Data (AtomicJ Repository)

This research utilized open-access simulated data for an elastic half-space with a Young’s modulus of E = 20 kPa and a Poisson’s ratio of v = 0.5. The data were generated using Mathematica 8.0, considering sphero-conical and blunted pyramidal indenters with radii of 0.1 μm and 0.2 μm, mounted on a cantilever with a spring constant of 0.1 N/m [25]. The synthetic data were created using the indenters described above, with random Gaussian noise added [25]. The force-indentation data have been deposited in the AtomicJ repository (https://sourceforge.net/projects/jrobust/files/TestFiles/, accessed on 1 August 2024) [25].

### 2.4. Open-Access Experimental Data (AtomicJ Repository)

Human prostate carcinoma DU-145 cells were cultured in DMEM-F12 HAM (Sigma, Saint Louis, MO, USA), supplemented with 10% fetal bovine serum and antibiotics, and maintained at 37 °C in a humidified atmosphere with 5% CO_2_. Force curves were obtained using an Agilent 5500 Atomic Force Microscope (Agilent Technologies, Santa Clara, CA, USA) equipped with silicon nitride cantilevers (Veeco Probes, Plainview, NY, USA) featuring a nominal tip radius of 20 nm, a half-angle of 25°, and a nominal spring constant of 0.01 N/m. The spring constant used to calculate Young’s modulus was determined via the thermal tune method. Measurements were conducted in culture medium at 37 °C. For data processing, only the first approximately 60 nm of each curve was used to ensure that h < 3R. The force-indentation data have been deposited in the AtomicJ repository (https://sourceforge.net/projects/jrobust/files/TestFiles/, accessed on 11 September 2024) [25].

### 2.5. Collagen Hydrogels

In this research, commercially available collagen hydrogels with predetermined stiffness were employed. We specifically used collagen hydrogels with a stiffness values of 1.0 kPa, which were adhered to 35 mm Petri dishes (Petrisoft 35mm, Cell Guidance Systems, Ltd., Maia Building, Babraham Bioscience Campus, Cambridge CB22 3AT, UK).

### 2.6. AFM Experiment

Atomic force microscopy (AFM) experiments were conducted using a commercial AFM system (5500, Keysight Technologies, Santa Rosa, CA, USA) equipped with V-shaped soft silicon nitride probes (MLCT-Bio, Cantilever D, Bruker). Due to the high water content of the sample, a Poisson’s ratio of ν = 0.5 was assumed. The spring constant was calibrated using the thermal noise method, while sensitivity calibration (measured as nanometers of cantilever deflection per volt signal from the laser detection system) was achieved by acquiring force-versus-distance curves on a Petri dish, which offered a pristine, rigid surface [26,27].

## 3. Results

**Figure 2:**
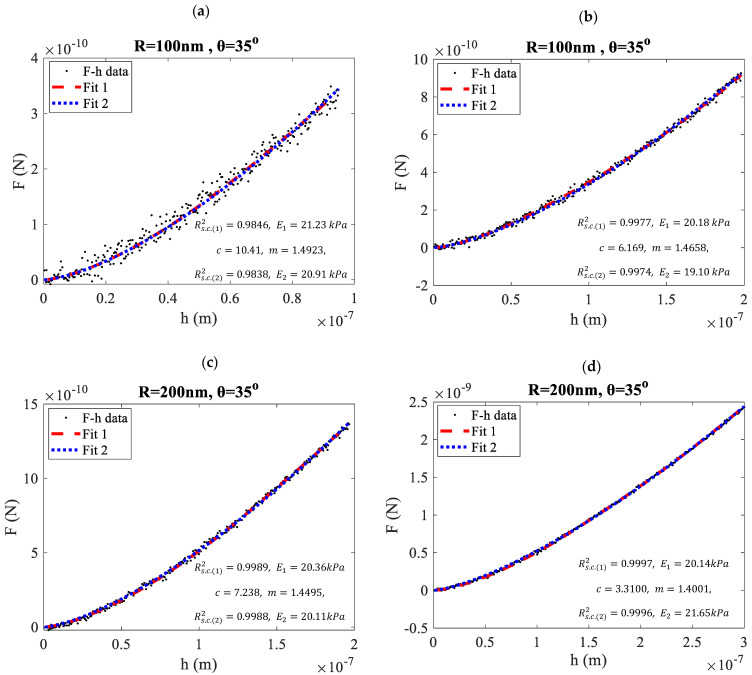
Fitting both classic and newly derived equations to simulated force-indentation data for various cases involving indentations with blunted indenters. (**a**) R = 100 nm, $\theta =35^{{}^{\circ}}$, $h_{max}=100\;nm$, (**b**) R = 100 nm, $\theta =35^{{}^{\circ}}$, $h_{max}=200\;nm$, (**c**) R = 200 nm, $\theta =35^{{}^{\circ}}$, $h_{max}=200\;nm$, (**d**) R = 200 nm, $\theta =35^{{}^{\circ}}$, $h_{max}=300\;nm$. ‘Fit 1’ refers to the fitting using classic equations (i.e., Equations (3)–(5) and (8)), while ‘Fit 2’ represents the proposed approach (Equations (2) and (17)). The Young’s moduli calculated using ‘Fit 1’ and ‘Fit 2’ are indicated in each figure as $E_{1}$ and $E_{2}$, respectively.

The accuracy of the proposed equation (Equation (17)) was further evaluated using open-access simulated data obtained with the protocol described in Section 2.3. In Figure 2, force-indentation data (F-h data) created by considering blunted conical indenters with $\theta =35^{{}^{\circ}}$ are shown. In Figure 2a,b the tip radius was R = 100 nm, while in Figure 2c,d, it was R = 200 nm. ‘Fit 1’ represents the classic approach (i.e., Equation (8) for a < b and Equations (3)–(5) for a > b), while ‘Fit 2’ represents a simple fit to Equation (2). Fitting to the classic Equations (3)–(5) and (8) was performed using AtomicJ software 2022 [25], while fitting to Equation (2) was done using MATLAB R21a. The Young’s modulus in the proposed approach (i.e., ‘Fit 2’) was calculated using Equations (10) and (17).

Equation (10) was used to determine the n-parameter (using the fitting parameter m), and Equation (17) was used to calculate the Young’s modulus (using the fitting parameter c). The fitted curves using both methods were accurate, since the R-squared coefficient ($R_{s.c.}^{2}$) was always close to 1. In every case, the Young’s moduli obtained using ‘Fit 1’ (denoted as $E_{1}$) and ‘Fit 2’ (denoted as $E_{2}$) were nearly identical (see Figure 2).

**Figure 3:**
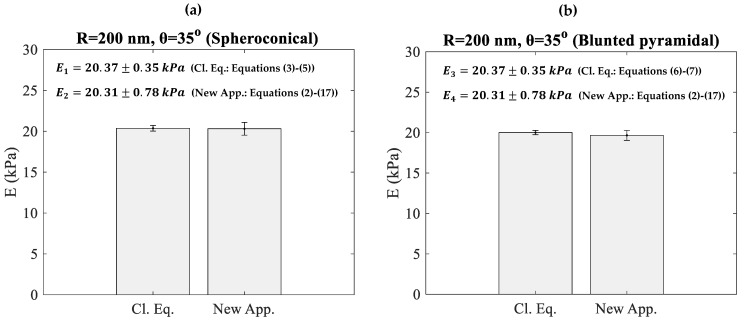
(**a**) A bar chart showing the Young’s moduli from 30 simulated force-indentation curves (using the protocol described in Section 2.3), calculated using the classic equations for sphero-conical indenters (Equations (3)–(5)) and the proposed approach (Equations (2) and (17)). The indenter’s radius was 200 nm, and its cone’s half-angle was 35°. $E_{1}$ denotes the Young’s modulus calculated using the classic approach (Equations (3)–(5)), and $E_{2}$ denotes the Young’s modulus calculated using the proposed Equations (2) and (17). (**b**) A bar chart showing the Young’s moduli from 30 simulated force-indentation curves (using the protocol described in Section 2.3), calculated using the classic equations for blunted pyramidal indenters with *R* = 200 nm and θ = 35° (Equations (6) and (7)) and the proposed approach (Equations (2) and (17)). $E_{3}$ denotes the Young’s modulus calculated using the classic approach (Equations (6) and (7)), and $E_{4}$ denotes the Young’s modulus calculated using the proposed Equations (2) and (17). ‘Cl. Eq.’ in the charts denotes the classic approach (Equations (3)–(7)), while ‘New App.’ refers to the method proposed in this paper (Equations (2) and (17)).

In addition, in Figure 3a, we present the Young’s moduli using a sphero-conical indenter (R = 200 nm, $\theta =35^{{}^{\circ}}$) for thirty additional simulated curves using both the classic approach ($E_{1}$) and the proposed method ($E_{2}$). ‘Cl. Eq.’ in the charts denotes the classic approach (Equations (3)–(5)), while ‘New App.’ refers to the method proposed in this paper (Equations (2) and (17)). When using the classic Equations (3)–(5), the mean value ± standard deviation resulted in $E_{1}=20.37\pm 0.35\;\mathrm{kPa}$, while when using the approach proposed by this paper, (Equations (2) and (17)) $E_{2}=20.31\pm 0.78\;\mathrm{kPa}$. The results using both methods are nearly identical.

In addition, simulated data (from thirty additional simulated force-indentation curves) using a 4-sided blunted pyramidal indenter with $\theta =35^{{}^{\circ}}$ and R = 200 nm are also presented in Figure 3b. The proposed method can be safely used for pyramidal indenters according to the results. When using the classic approach for pyramidal indenters (Equations (6) and (7)), the mean value ± standard deviation resulted in $E_{3}=20.01\pm 0.27\;\mathrm{kPa}$. In addition, when using the proposed approach (Equations (2) and (17)), the Young’s modulus resulted in $E_{4}=19.66\pm 0.61\;\mathrm{kPa}$. The results are also similar in this case.

**Figure 4:**
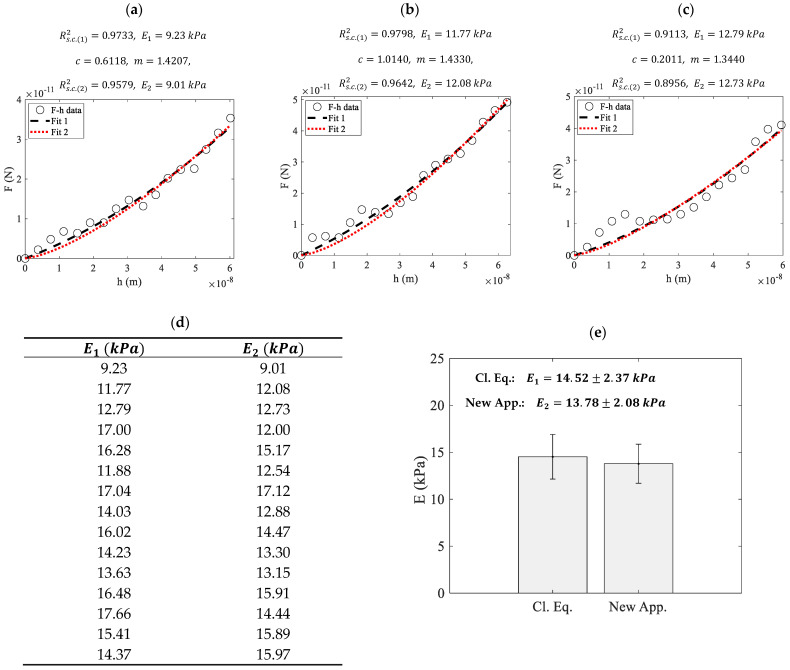
(**a**–**c**) Force-indentation data from prostate cancer cells and fitted curves. Fit 1 denotes a fit to the classic Equations (3)–(5), while Fit 2 denotes the fit to Equations (2) and (17). The classic approach and the simplified method yielded similar results. (**d**) Young’s modulus values determined from 15 force-indentation curves using the aforementioned methods. (**e**) A bar-chart using the data shown in (**d**). ‘Cl. Eq.’ in the charts denotes the classic approach (Equations (3)–(5)), while ‘New App.’ refers to the method proposed in this paper (Equations (2) and (17)).

The method proposed in this paper was further tested on open-access experimental data on prostate cancer cells, following the protocol described in Section 2.4. In Figure 4a–c, characteristic force-indentation data are presented. The data were first fitted to the classic Equations (3)–(5) (Fit 1) and subsequently to Equations (2) and (17) (Fit 2). $E_{1}$ and $R_{s.c.(1)}^{2}$ denote the Young’s modulus and the R-squared coefficient when using the classic Equations (3)–(5), while $E_{2}$ and $R_{s.c.(2)}^{2}$ denote the same quantities when using Equations (2) and (17).

In addition, Figure 4d presents the Young’s moduli calculated from 15 force-indentation curves using the aforementioned methods. The differences in the results provided by the two methods are minimal in each case. The 15 measurements obtained using each of the methods described above (i.e., the classic method resulting in $E_{1}$ and the proposed method resulting in $E_{2}$) are presented comparatively in the bar chart in Figure 4e. For the first case, $E_{1}=14.52\pm 2.37\;\mathrm{kPa}$ and for the second case, $E_{2}=13.78\pm 2.08\mathrm{kPa}$. The fitting parameters *c* and *m* are also presented. The results are similar in each case.

## 4. Discussion

In this paper, a new equation for processing force-indentation data using real indenters was presented. The equation was derived using simple mathematical and physics-based criteria, and its major advantage is that it directly relates the applied force to the indentation depth. The proposed equation demonstrated excellent accuracy for small indentation depths. The approach for determining Young’s modulus is straightforward: the force-indentation data are fitted to Equation (2). Subsequently, Young’s modulus is calculated using Equations (10) and (17), provided that the geometrical features of the AFM tip are known.

An interesting point for discussion is the value of the exponent *m* when the data are fitted to Equation (2) for indentation depths comparable to the tip radius. According to classic approaches presented in [20,21], when using blunted conical or pyramidal indenters, the data should be fitted to Equation (8) for a < b, and to Equations (3)–(5) (for a blunted conical shape) or Equations (6) and (7) (for a blunted pyramidal shape).Therefore, it is reasonable to expect that the exponent m should be in the range 1.5 < m < 2 (since for small indentation depths m ≅ 3/2 (Equation (8)), while for large indentation depths m ≅ 2 (Equation (1)). Conversely, the exponent m may become significantly smaller than m = 3/2 (e.g., Figure 1a). This is because the spherical parts of the indenter are only approximately described by Equation (8). Equation (8) is accurate for parabolic indenters and can also be used for spherical indenters, but only for small indentation depths relative to the tip radius. Therefore, Equation (8) cannot accurately describe the initial stages of indentation when the spherical parts of the indenter are in contact with the sample. The accurate equation for spherical indentations is presented below [28]:

(20) $$F=\frac{4ER^{\frac{1}{2}}}{3\left(1-v^{2}\right)}h^{\frac{3}{2}}Z\;$$

where

(21) $$Z=c_{1}+\sum_{M=2}^{N}\frac{3}{2M}c_{M}R^{\left(\frac{3}{2}-M\right)}h^{M-3/2}\;$$

For h<R, N = 3 and $c_{1}$ = 1.014, $c_{2}=-0.09059$ and $c_{3}=-0.09431$ [28]. Therefore,

(22) $$Z=c_{1}+\frac{3}{4}c_{2}R^{-\frac{1}{2}}h^{\frac{1}{2}}+\frac{3}{6}c_{3}R^{-\frac{3}{2}}h^{\frac{3}{2}}\;$$

Equation (20) can be fitted to Equation (2) for 1<m<1.5 [29]. Therefore, the spherical portion of the indenter for indentation depths comparable to the tip radius results in small m-coefficients. An important conclusion from the analysis presented above is that the exponent *m* can vary significantly depending on the indentation depth, even when using the same indenter. For very small indentation depths (i.e., $h_{max}\ll R$), Z→1, and the exponent *m* equals 3/2 (this is the classic Hertzian equation valid for parabolic or spherical indenters at low indentation depths). For larger indentation depths, where $h_{max}<h_{T}$ ($h_{T}$ is the transition depth between the spherical and the conical parts of the indenter), the exponent *m* decreases. It has been theoretically proven in [28] and experimentally validated in [30] that for spherical indentations, as $h_{max}\to R$, the force-indentation data tend toward a linear relationship [28]. In addition, for $h_{max}\gg R$, the sphero-conical indenter can be approximated as a perfect cone, thus m→2. Therefore, depending on the indentation depth, the exponent *m* can vary significantly in the range 1<m≤2, based on the shape of the indenter parts that are in contact with the sample. There are two ‘mistakes’ that can lead to an incorrect calculation of Young’s modulus. The first mistake is using the exponent m=3/2 for spherical indenters (or spherical parts of the indenter) regardless of the indentation depth. This approximation can lead to errors in the range of approximately 30–40% [28]. In this case, the Young’s modulus will be underestimated, because Z < 1. The second mistake is considering a conical shape for blunted pyramidal indenters. In this case, the Young’s modulus is significantly overestimated, with errors potentially reaching 100% [22].

**Figure 5:**
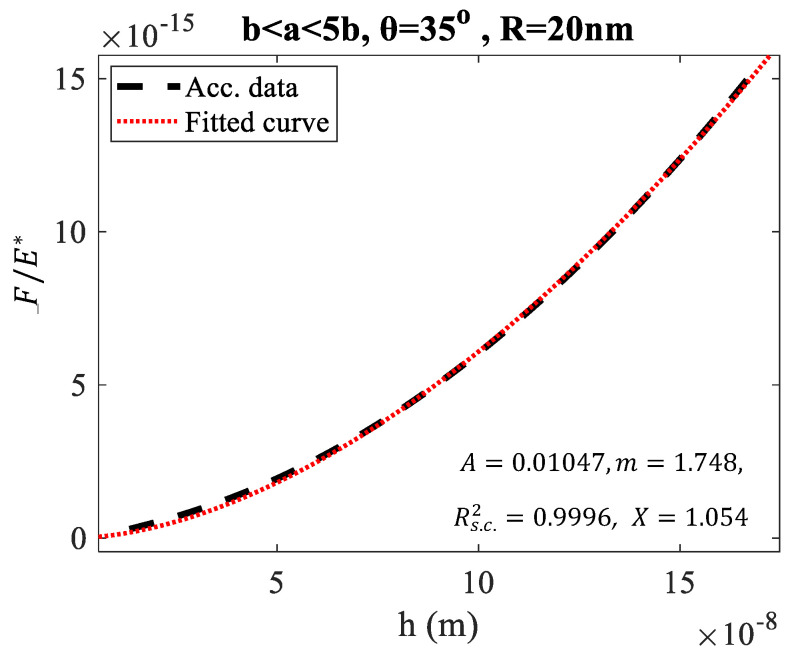
The force-indentation data for a theoretical case in the domain b < a < 5b (16.4 nm < a < 81.9 nm) using a sphero-conical indenter with $\theta =35^{{}^{\circ}}$ and R = 20 nm are presented. In this case, $h_{max}$/R = 8.35, and Equation (17) yields approximately a 5% error in the calculation of Young’s modulus. Equation (17) provides accurate results for h < 3R.

In addition, it is important to discuss the limitations of Equation (17). It was designed to be accurate for shallow indentations (e.g., Figure 1 and Figure 2). It can be safely used to model the force-indentation data for h < 3R. For h > 3R, it yields small errors. For example, in Figure 5, the $\frac{F}{E^{*}}=f(h)$ data for the case of a sphero-conical indenter with R = 20 nm and $\theta =35^{{}^{\circ}}$ in the domain b < a < 5b (where $\frac{h_{max}}{R}=8.35$) is presented. In this case, X=1.054 (i.e., a 5.4% difference compared to the actual value). At this point, it is important to note that this difference should not be compared to the minimal differences presented in Figure 3 and Figure 4. For example, in Figure 3, the differences relative to the actual value of 20 kPa were due to random Gaussian noise in the simulations, which was added to simulate real conditions. In contrast, in the case of Figure 5, the difference arises between the actual value (as calculated using the classic Equations (3)–(5)) and the value calculated using the approximate Equation (17). For small h/R ratios, this difference is very small, as shown in Figure 1.

It is also important to note that there is no need to search for simpler, more accurate solutions for larger indentations (i.e., to minimize the approximately 5% error), as an alternative approach has recently been developed and proven to be accurate for h > 3R [22]. For large indentation depths, the transition depth can be approximated to $h_{T}\approx R$ [22]. Therefore, the Young’s modulus can be determined using the equations below [22]:

(23) $$F=\frac{1}{m}\frac{2Er_{c}}{1-v^{2}}h\;$$

where

(24) $$r_{c}\approx \frac{2}{\pi }htan\left(\theta \right)+\frac{2}{\pi }R\left[1-\tan\left(\theta \right)\right]$$

Therefore, for h<3R, a straightforward equation between F, h is Equation (12), while for h > 3R, Equations (23) and (24) should be used instead.

**Figure 6:**
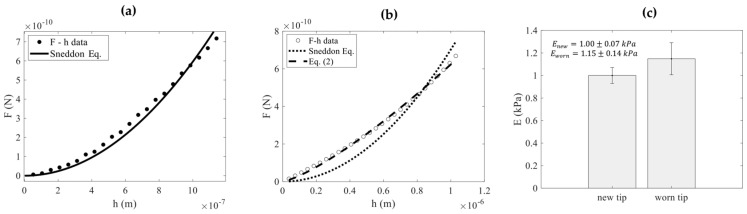
Force-indentation data on a collagen hydrogel with predefined stiffness (E = 1 kPa) using an MLCT-Bio tip (cantilever D). (**a**) The force-indentation data follow Equation (1) when using a ‘new’ undamaged tip. (**b**) The force-indentation data on the same hydrogel after multiple experiments in various areas. Due to severe tip damage, the data follow Equation (2) instead of Equation (1). (**c**) The results obtained from 10 force-indentation curves using a ‘new’ tip and 10 curves after ‘extensive use of the tip’ are presented comparatively.

Another critical aspect to discuss is the need for deriving simple equations for data processing in AFM experiments. It is important to note that in most cases where sharp pyramidal tips are employed, Sneddon’s equation is used for data processing [31]. However, in many cases, the validity of Equation (1) (i.e., Sneddon’s equation), even when a sharp pyramidal tip is used, is questionable. For example, characteristic force-indentation data on collagen hydrogels with predefined stiffness (E = 1 kPa) are shown in Figure 6. The data were fitted to Sneddon’s equation (Equation (1)), resulting in $F=595.8h^{2}\;(S.I.)$ (Figure 6a). The R-squared coefficient resulted in $R_{s.c.}^{2}=0.9781$. The Young’s modulus resulted in E=1.003 kPa as expected. Figure 6b presents force-indentation data collected after a large number of experiments (imaging in contact mode and indentation) using the same AFM tip. The force-indentation data do not follow Sneddon’s equation due to tip damage [32]. The fit to Sneddon’s equation ($F=702.2h^{2}\;(S.I.)$) was poor; $R_{s.c.}^{2}=0.9120$. The Young’s modulus value using Equation (1) resulted in E=1.18 kPa. It is also noteworthy that the data in Figure 6b perfectly follow Equation (2) with $c=0.0367\;Newtons\times meters^{-1.295}$ and m=1.295 as $R_{s.c.}^{2}=0.9976$. A rational explanation of these values is severe damage or contamination of the tip. For example, a ‘flattened’ tip apex due to significant wear may result in a small m-coefficient, as the force-indentation data remain linear when the contact area is constant [28,30]. It is interesting to note that, despite the small indenter tip radius compared to the maximum indentation depth (the nominal tip radius is 20 nm while the maximum indentation depth is 1000 nm), which allows for a perfect conical approximation in the first experiment (Figure 6a), the tip damage can be severe enough to cause significant deviations from the conical model [32].

**Figure 7:**
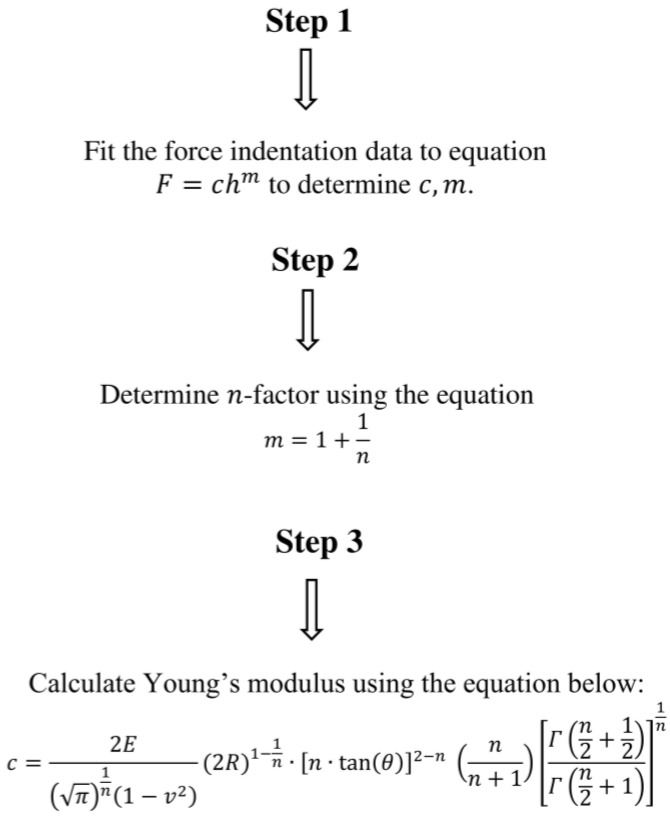
A flowchart of the proposed method.

In Figure 6c, the results obtained from 10 force-indentation curves using a ‘new’ tip and 10 curves after ‘extensive use of the tip’ are presented comparatively. Sneddon’s equation is no longer appropriate for data processing. In this case, a new calibration of the tip is required, and subsequently, Equation (23) is more suitable for data processing. This example highlights the need for developing new mathematical models to account for the accurate shape of the AFM tip. In addition, a flowchart of the proposed method is presented in Figure 7.

It is also important to note that shallow indentations are mandatory in many cases when using pyramidal indenters. For example, when testing thin materials such as collagen fibers [33,34,35,36,37,38,39,40], the maximum indentation depth is comparable to the indenter’s radius. Otherwise, there will be a significant overestimation of the Young’s modulus due to substrate effects [41,42,43]. In addition, when testing biological cells, the apparent Young’s modulus decreases as the indentation depth increases, approaching a limit value [44,45,46]. The reason for this behavior is related to the mechanical heterogeneity of cells [44,46] and the surface tension [45]. Force-indentation data on cells are usually fitted to Sneddon’s equation (Equation (1)) [32,44,46,47]. However, a non-negligible tip radius can lead to an overestimation of the Young’s modulus as already mentioned [48]. Therefore, the true depth-dependent mechanical properties at the initial stages of indentation will not be determined. Therefore, Equation (17) is appropriate for modeling the initial portion of the force-indentation data in all cases. An additional question to be answered is whether Equation (17) is valid under a mathematical perspective for heterogeneous materials. The answer is positive. Consider a highly heterogeneous material that is being tested with a blunted conical or pyramidal indenter. In this case, the Young’s modulus should be strongly depth-dependent. To characterize this heterogeneous material, we can consider an equivalent experiment with the same indenter, the same indentation depth, and the same work done by the indenter (i.e., the area under the force-indentation curve) [46]. In this case, the fitting parameter yields the average Young’s modulus of the sample for a specific domain. A rigorous mathematical approach for further clarification is presented below. By combining Equations (10), (17) and (23), it is concluded that

(25) $$r_{c}=\frac{1}{{\left(\sqrt{\pi }\right)}^{\frac{1}{n}}}(2{R)}^{1-\frac{1}{n}}\cdot {\left[n\cdot \tan\left(\theta \right)\right]}^{2-n}{\left[\frac{\Gamma \left(\frac{n}{2}+\frac{1}{2}\right)}{\Gamma \left(\frac{n}{2}+1\right)}\right]}^{\frac{1}{n}}h^{\frac{1}{n}}\;$$

The contact stiffness is provided below [49,50,51]:

(26) $$S=\frac{dF}{dh}=2E^{*}\left(h\right)r_{c}\left(h\right)\;$$

In Equation (26), it is assumed that the material’s properties are depth-dependent. The applied force on the sample is calculated as follows:

(27) $$F=\int_{0}^{h}Sdy=2\int_{0}^{h}E^{*}\left(y\right)r_{c}\left(y\right)dy\;$$

The next step is to used the weighted mean value theorem for integrals [52]. Let $E^{*}$, $r_{c}$: [0, ℎ]→*R* be such that $E^{*}$ is continuous and $r_{c}$ is integrable and does not change the sign on [0, *h*]. Then, there exists a number *c*_0_∈(0, ℎ) such that

(28) $$F=2E^{*}\left(c_{0}\right)\int_{0}^{h}r_{c}\left(y\right)dy\;$$

Therefore,

(29) $$F=\frac{2E^{*}(c_{0})}{{\left(\sqrt{\pi }\right)}^{\frac{1}{n}}}(2{R)}^{1-\frac{1}{n}}\cdot {\left[n\cdot \tan\left(\theta \right)\right]}^{2-n}\;\left(\frac{n}{n+1}\right){\left[\frac{\Gamma \left(\frac{n}{2}+\frac{1}{2}\right)}{\Gamma \left(\frac{n}{2}+1\right)}\right]}^{\frac{1}{n}}h^{1+\frac{1}{n}}\;$$

The parameter $E^{*}(c_{0})$ represents the average properties of the material in the domain 0≤y≤h. Therefore, the procedure described in this paper can be applied in all cases. However, it should be noted that the average Young’s modulus will strongly depend on the indentation depth (i.e., the result will differ if the indentation depth is h = R or h = 1.5R).

In addition, it should be also noted that uncertainties in determining deflection sensitivity and the cantilever’s spring constant are significant sources of error in AFM experiments [53]. For instance, Matei et al. reported that the precision and accuracy of the thermal method are 5% and 10%, respectively [54]. Additionally, Schillers et al. noted that the error in deflection sensitivity for cell samples can range from 5% to 20%. These errors are unrelated to the geometrical features of the AFM tip examined in this paper. To minimize these errors when testing soft materials, such as cells, a reliable technique called the Standardized Nanomechanical AFM Procedure (SNAP) has been developed [53]. SNAP facilitates precise adjustment of the AFM optical lever system—a vital requirement for all force spectroscopy methods—yielding reliable values that remain consistent across different instruments, laboratories, and operators. However, the uncertainties mentioned above are the main reason our equations were initially tested on simulated curves in this paper: the focus was placed entirely on the geometrical features of the indenters, excluding any other sources of error that could potentially affect our results.

## 5. Conclusions

In this paper, a new, simple equation for data processing in force-indentation experiments using blunted conical or pyramidal indenters is presented. The proposed equation is an ideal simple alternative for h < 3R. The accuracy of this approach was validated using simulated force-indentation experiments with blunted indenters and experimental data on prostate cancer cells. The proposed equation is particularly effective for analyzing the initial portion of force-indentation curves or for shallow indentations (e.g., to avoid substrate effects) and will be a valuable tool for streamlining data processing in AFM experiments. This simplification in AFM data processing represents a significant step forward in applying AFM methods in real clinical procedures. The results of this paper can be applied to streamline the data fitting process in AFM indentation experiments on soft samples. More particular, new software can be developed to fit the data in the trivial Equation (2) in all cases. Four options will be provided to the user. For parabolic or spherical indentations with a small ℎ/*R* ratio (h/R < 0.1), *m* = 3/2, and the Young’s modulus will be calculated using the fitting constant *c*, where $c=\frac{4}{3}\frac{E}{1-v^{2}}R^{1/2}$. For pyramidal tips and high ℎ/*R* ratios (i.e., h/R >> 1; for example, a typical limit should be h/R > 10), m=2, and the Young’s modulus can be calculated using $c=\frac{2}{\pi }\frac{E}{1-v^{2}}\tan(\theta )$. For ‘intermediate’ cases (under the condition h < 3R), the exponent *m* will be calculated by the software along with the parameter c. In this case, the Young’s modulus will be determined using Equation (17). Lastly for h > 3R, Equations (23) and (24) will be employed, according to the analysis presented in [22].

## Data Availability

The original contributions presented in the study are included in the article, further inquiries can be directed to the corresponding author.
